# Environmental and human iodine and selenium status: lessons from Gilgit-Baltistan, North-East Pakistan

**DOI:** 10.1007/s10653-021-00943-w

**Published:** 2021-05-07

**Authors:** Saeed Ahmad, Elizabeth H. Bailey, Muhammad Arshad, Sher Ahmed, Michael J. Watts, Alex G. Stewart, Scott D. Young

**Affiliations:** 1 Division of Agricultural and Environmental Sciences, School of Biosciences, University of Nottingham, Sutton Bonington Campus, Loughborough, LE12 5RD Leicestershire UK; 2 Mountain Agriculture Research Centre Gilgit (Pakistan Agricultural Research Council), Gilgit-Baltistan, Pakistan; 3 Centre for Environmental Geochemistry, Inorganic Geochemistry, British Geological Survey, Nottingham, NG12 5GG UK; 4 College of Life and Environmental Science, University of Exeter, Exeter, EX4 4RJ UK

**Keywords:** Environmental iodine and selenium, Iodised salt, Diet, Urine, Hydration correction, Pakistan

## Abstract

Iodine and selenium deficiencies are common worldwide. We assessed the iodine and selenium status of Gilgit-Baltistan, Pakistan. We determined the elemental composition (ICP-MS) of locally grown crops (*n* = 281), drinking water (*n* = 82), urine (*n* = 451) and salt (*n* = 76), correcting urinary analytes for hydration (creatinine, specific gravity). We estimated dietary iodine, selenium and salt intake. Median iodine and selenium concentrations were 11.5 (IQR 6.01, 23.2) and 8.81 (IQR 4.03, 27.6) µg/kg in crops and 0.24 (IQR 0.12, 0.72) and 0.27 (IQR 0.11, 0.46) µg/L in water, respectively. Median iodised salt iodine was 4.16 (IQR 2.99, 10.8) mg/kg. Population mean salt intake was 13.0 g/day. Population median urinary iodine (uncorrected 78 µg/L, specific gravity-corrected 83 µg/L) was below WHO guidelines; creatinine-corrected median was 114 µg/L but was unreliable. Daily selenium intake (from urinary selenium concentration) was below the EAR in the majority (46–90%) of individuals. Iodine and selenium concentrations in all crops were low, but no health-related environmental standards exist. Iodine concentration in iodised salt was below WHO-recommended minimum. Estimated population average salt intake was above WHO-recommended daily intake. Locally available food and drinking water together provide an estimated 49% and 72% of EAR for iodine (95 µg/day) and selenium (45 µg/day), respectively. Low environmental and dietary iodine and selenium place Gilgit-Baltistan residents at risk of iodine deficiency disorders despite using iodised salt. Specific gravity correction of urine analysis for hydration is more consistent than using creatinine. Health-relevant environmental standards for iodine and selenium are needed.

**Supplementary Information:**

The online version contains supplementary material available at 10.1007/s10653-021-00943-w.

## Introduction

Iodine (I) and selenium (Se) are important micronutrients for human health and play a vital role in the synthesis of thyroid hormones and wider aspects of metabolism, including brain development in early pregnancy (Jin et al., 2019; Schomburg & Köhrle, 2008; Velasco et al., 2018; Wu et al., 2015). Low dietary I causes a variety of health complications, collectively known as iodine deficiency disorders (IDD) (Hetzel 1983; Zimmermann 2009), which are some of the most important and common preventable public health problems worldwide (Kapil, 2007; Gizak et al., 2017; IGN, 2020a). Iodine deficiency can affect any stage of human development including foetus, neonate, child, adolescent and adult (Hetzel, 2000; Zimmermann et al., 2008). However, the consequences are more serious during foetal development and childhood (Bath et al., 2013; Hay et al., 2019; Obican et al., 2012; Velasco et al., 2018) with pre-pregnancy deficiency thought to be deleterious to brain development (Velasco et al., 2018). Globally, approximately two billion people are living at risk of IDD, including 240–285 million school-age children (Anderson et al., 2007; Biban & Lichiardopol, 2017; IGN, 2020b). Iodine deficiency was widespread in Pakistan prior to the start of the Universal Salt Iodisation programme in 1994, with goitre prevalence of > 70% in some parts of the country (Khattak et al., 2017). It is reported that the Universal Salt Iodisation programme has resulted in up to a 50% decrease in IDD, but it is suspected that this decline may be non-uniform and possibly over-estimated (Khattak et al., 2017). Our study area, Gilgit-Baltistan in the remote mountainous north-east, has long been known for endemic IDDs (Chapman et al., 1972; McCarrison, 1909; Stewart, 1990), which are still considered a major public health problem there (Shah et al., 2014). The 2011 national nutritional survey of Pakistan reported a urinary I concentration (UIC) of 64 and 68 µg/L in mothers (*n* = 34) and 6–12-year-old children (*n* = 29) from Gilgit-Baltistan (GoP, 2011), well below the adequate range of 100–199 µg/L, and classed as mild iodine deficiency by WHO (Benoist et al., 2004), although the sample size was small.

Selenium deficiency is associated with a range of health problems including cardiovascular and myodegenerative diseases, infertility and cognitive decline (Rayman, 2000, 2012; Shreenath & Dooley, 2019). There are approximately 0.5–1.0 billion people affected by Se deficiency globally (Haug et al., 2007; Jones et al., 2017). A coexisting deficiency of both I and Se aggravates hypothyroidism and may result in myxoedematous cretinism and other thyroid disorders (Arthur et al., 1999; Rayman, 2000; Ventura et al., 2017). Currently, there appears to be no literature available on the Se status of the Gilgit-Baltistan population.

Both I and Se deficiencies occur when there is inadequate dietary intake of these essential elements (Bath et al., 2014; Kapil, 2007; Rayman, 2008; Shreenath & Dooley, 2019). The I nutritional status of a population can be estimated from UIC, which is a well-established, cost-effective and easily obtainable biomarker (WHO, 2013). Selenium concentration in blood plasma is the trusted biomarker for assessing Se level at both individual and population level (Fairweather-Tait et al., 2011), but obtaining blood plasma samples is demanding. However, urinary Se concentration (USeC) has been reported as a plausible alternative to blood plasma to assess population-level Se status (Middleton et al., 2018; Phiri et al., 2020).

The population of Gilgit-Baltistan consumes mainly locally grown foods. Therefore, we aimed to (1) measure I and Se concentration in locally grown agricultural produce, (2) evaluate the I and Se status of residents of the Gilgit district of Gilgit-Baltistan using urinary concentrations (UIC, USeC) with corrections for hydration, and (3) study the role of iodised salt in the I status of the local population using urinary sodium concentration as an indicator of salt intake.

## Methods

### Sampling overview

Two separate sets of samples were collected: (1) environmental samples comprising edible plants and drinking water, and (2) population samples including individual urine samples, and family-based culinary salt samples supported by a salt intake questionnaire.

**Fig. 1:**
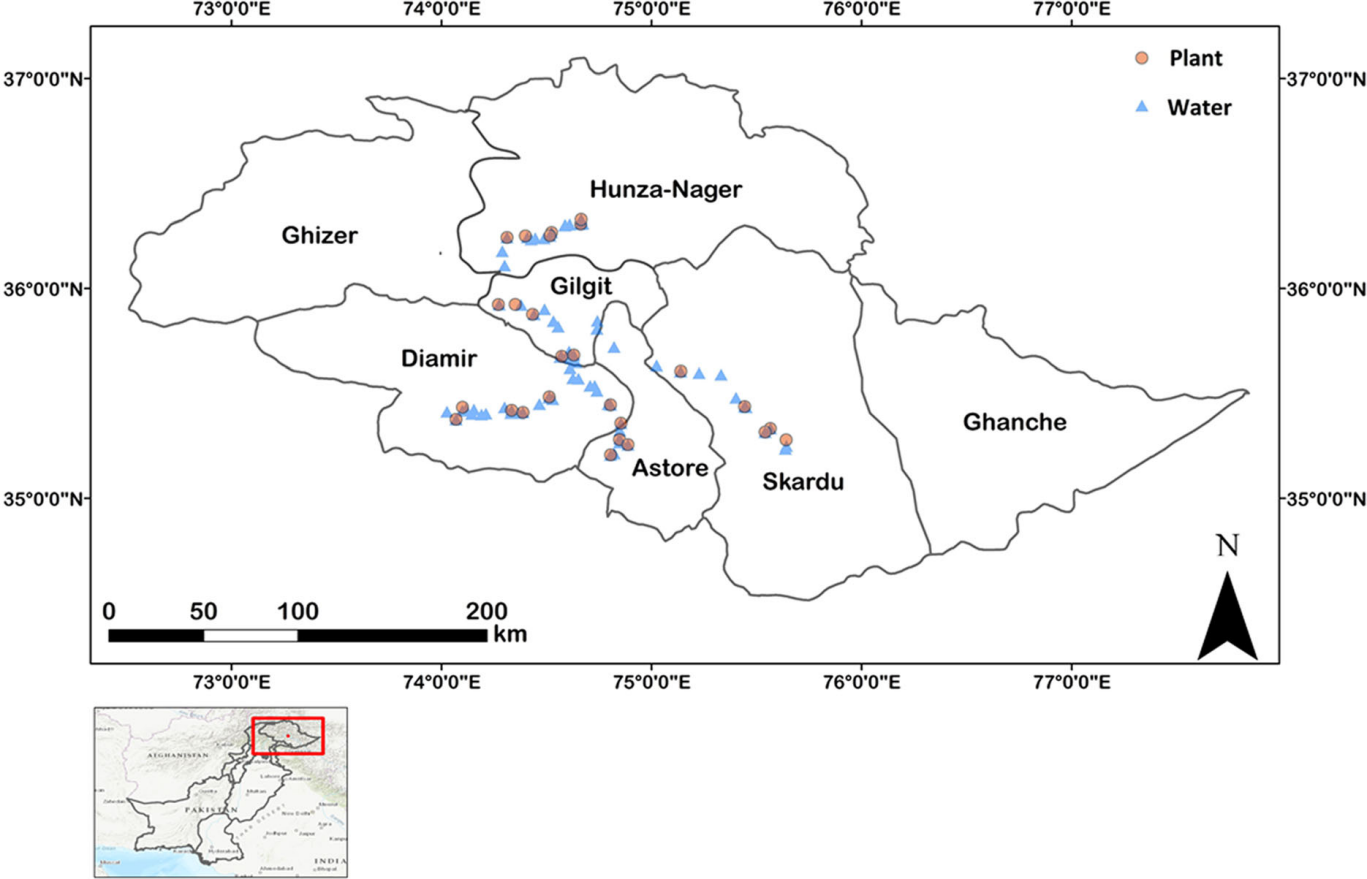
Locations of plant and water samples in Gilgit-Baltistan

A wide range of locally grown edible plants (*n* = 281), representing 34 different crop types from various villages across five districts (Fig. 1) of Gilgit-Baltistan, covering approximately 65% of the food items consumed daily, were sampled. The selection of villages for plant sampling was part of a wider study that included soil and irrigation water sampling (Ahmad et al., 2021) and for which the criteria for selection was the accessibility and availability of fertile agricultural land. Mountain Agriculture Research Centre (MARC) staff, who are familiar with the area and have multiple research stations across different districts of Gilgit-Baltistan, identified sampling villages. Accessibility in our sampling design was important because Gilgit-Baltistan is very mountainous with rough terrain and some villages are difficult to reach. Water was sampled in the villages where plant sampling was undertaken and also between villages (Fig. 1). Samples (*n* = 82) were collected from a mix of surface water (rivers/streams/lakes) and groundwater (springs/wells), all of which were used as drinking water by the Gilgit-Baltistan local population throughout the year.

The University of Nottingham Research Ethics Committee granted ethical approval for (1) urine sampling of individuals representing different age groups and (2) undertaking a household questionnaire survey to estimate local salt consumption. MARC staff obtained verbal permission from the elders of the community; informed written consent from each adult and the parents of each participating child was also obtained. For the urine sampling, the study was advertised to the wider population of the district of Gilgit only, with the support of MARC staff and community elders by verbal announcements and notices in community centres. Then, our survey team organised community gatherings and provided verbal and written information about the study to the local population to recruit volunteers. Only healthy local volunteers (*n* = 415), representing different age groups and gender, who declared no known history of medically diagnosed thyroid disease were recruited to participate in the study. The participants came from 76 different households; therefore, a single salt sample was obtained from each participating household. Furthermore, a female representative of each household, who was responsible for cooking and other food preparations for the household, was asked to attend a face to face interview and answer a standard salt intake questionnaire.

### Sample preparation

#### Plants

Plants were oven-dried at 40 °C and then milled in an ultra-centrifugal mill to obtain finely ground powder for analysis by ICP-MS (iCAP-Q; Thermo-Fisher, Bremen, Germany). Approximately 0.2 g of the finely ground plant sample was microwave acid digested in 6 mL of 70% HNO_3_ in a pressurised PFA vessel at 140 °C. Grain samples were digested in 3 mL HNO_3_ (70%), 3 mL Milli-Q water and 2 mL H_2_O_2_ (30%). Sample digests of plants and grain were diluted to 2% HNO_3_ prior to Se analysis by ICPMS. Microwave-assisted tetra methyl ammonium hydroxide (TMAH) extraction of plant samples (c. 0.2 g) was undertaken at 110 °C in 5 mL of 5% TMAH. The extracts were diluted to 1% TMAH with Milli-Q water, centrifuged, syringe-filtered to < 0.22 µm and analysed by ICPMS.

#### Water

Water samples were filtered at the sampling point, using syringe filters (< 0.22 µm), into a universal tube and kept in the dark at 4 °C. Duplicate samples were adjusted to 1% TMAH or acidified to 2% HNO_3_ prior to I and Se analysis, respectively.

#### Urine

A first-morning void spot urine sample was collected in an 8-mL capacity polypropylene tube from each participant, kept in a cool box without filtration and transported to the MARC laboratory where they were stored at − 20 °C. Frozen urine samples were shipped to the University of Nottingham for elemental and hydration adjustment analyses.

#### Salt

Upon collection in a sealed plastic bag from each participating household, domestic salt samples were kept in the dark at the MARC in Gilgit before shipping to the University of Nottingham where they were stored at 20 °C pending chemical analysis.

### Salt intake survey

We designed a questionnaire specifically aimed at ascertaining information on the frequency and amount of salt (iodised/non-iodised) purchased, its storage and daily consumption. It was piloted on ~ 20% (*n* = 15) of surveyed households before the study began. The pilot survey results were excluded from the final analysis. The survey team members were fluent in speaking the local language (Shina) and were familiar with local culture which negated any barrier to communicating with the residents. Individual daily salt consumption was estimated by dividing the estimated amount of salt purchased by the number of individuals (age > 5 years) in a household allowing for the frequency of purchase.

### Chemical analysis

#### Plant and water samples

Analysis of I in all plant and water samples was undertaken in 1% TMAH with the ICP-MS operating in standard mode (no cell gas) and an internal standard of 5 µg/L rhenium (Re) to correct for instrumental drift. For Se analysis in an acidic matrix (2% HNO_3_), the ICP-MS (iCAP Q) instrument was in ‘hydrogen cell’ mode with internal standards of 5 µg/L rhodium (Rh) and Re in 4% methanol and 2% HNO_3_.

#### Salt samples

Duplicate samples (1.25 g) were dissolved in 25 mL of Milli-Q water and diluted 1-in-10 with 1% TMAH prior to I analysis by ICP-MS.

#### Urine samples

Elemental analyses (I, Se and Na) were made on all urine samples using ICP-MS. Iodine and Se were measured to confirm population I and Se status while Na was measured to estimate salt intake. Iodine was measured in an alkaline matrix following a 1-in-20 dilution (0.5 mL urine + 9.5 mL 1% TMAH), whereas Se and Na were measured in an acidic matrix (0.5 mL + 9.5 mL 2% HNO_3_).

About 90% of Na consumed is excreted in urine (McLean, 2014). Accordingly, participants’ salt intake was calculated using the method of Chen et al. (2018), who used 24 h urine samples to estimate Na intake. However, we used spot urine samples as these are a plausible alternative in determining population salt intake (Huang et al., 2016).

Adjustment of urinary analyte concentrations was undertaken both with urinary creatinine concentration and specific gravity to allow for the effect of hydration status. Urinary creatinine was measured spectrophotometrically, and specific gravity was measured with a handheld pocket refractometer (PAL-10S, Atago, Japan) on 300 µL of urine (Phiri et al., 2020; Watts et al., 2019).

Equation 1 was used to adjust urinary analyte concentration for variation in creatinine concentration.

1 $$C_{{\text{cre - adj}}} = \frac{{C_{m} }}{{C_{{{\text{cre}}}} }}$$

where *C*_cre-adj_ is the adjusted concentration (µg/g), *C*_*m*_ is the uncorrected concentration (µg/L) of analyte in urine and *C*_cre_ represents creatinine concentration (g/L). For each sample, correction with specific gravity was also undertaken, using Eq. 2.

2 $$C_{{\text{sg - adj}}} = C_{{m{ }}} \times \frac{{\left( {{\text{sg}}_{{{\text{mean}}}} - 1} \right)}}{{({\text{sg}}_{{{\text{meas}}}} - 1)}}$$

where *C*_sg-adj_ is the adjusted concentration (µg/L), sg_mean_ represents the average specific gravity of all the samples analysed in the study and sg_meas_ is the measured specific gravity.

### Calculating iodine and selenium dietary intake

We estimated the individual daily I and Se intake from locally grown food using typical consumption patterns from the Pakistani national food basket as no local food basket has been published. For the food types which were not sampled in this study but were part of the food basket, we obtained I and Se concentrations from published literature. As an alternative approach to estimating daily I and Se intake, we assumed that the entire diet originated locally and simply scaled up to 100% the 65% for which we could account from the survey data. Individual I and Se intake from drinking water was calculated based on a drinking water consumption of 2–4 L/day. The recommended value for adults and children is to drink at least 2 L water per day, but individual’s daily drinking water requirements vary from 2 to 4 L/day depending on the nature of their work and lifestyle and the climate of the area (WHO, 2016).

### Estimating iodine and selenium nutrition from urine

We used the WHO UIC epidemiological criteria (Zimmermann & Andersson, 2012; WHO, 2013) for evaluating population I nutrition and severity of I deficiency. There are no approved guidelines for estimating Se nutrition status from USeC, and different approaches are reported. We used three different reported approaches to estimate population Se nutrition status from USeC: (1) 50–70% of dietary Se is excreted in urine (Alaejos & Romero, 1993), (2) 40–60% of Se intake is excreted in urine (EFSA NDA, 2014), (3) 73 and 77% of ingested Se is excreted via urine in men and women, respectively (Nakamura et al., 2019; Yoneyama et al., 2008). We used a published age group classification (IOM, 2000) to describe Se nutrition.

### Quality control

Certified reference materials (CRM) wheat flour (NIST 1567b) and tomato leaves (NIST 1573a) were digested/extracted alongside plant samples. For I analysis in urine samples, two reference materials (Seronorm™ Trace Elements Urine, L-1 and L-2) were analysed simultaneously with urine samples.

### Statistical analysis

Statistical calculations (mean, median, standard deviation and interquartile ranges) were performed in Microsoft Excel 2016 while Minitab (version 18.1) was used for the Anderson Darling normality test, Pearson correlation and ANOVA.

## Results

### Population demographics

Of the sampled population (*n* = 415) (Table 1), 44% were male and 56% female; 32% were women of reproductive age (15–49 years), of whom 10% (*n* = 13) were reported to be pregnant (*n* = 7) and/or lactating (*n* = 7). Although the number of pregnant and nursing women is small, we cannot be sure that they are the only pregnant or nursing women in the study, so we have included them in the wider group of women of reproductive age.

**Table 1:** Number (*n*) and proportion (%) of each age group in the population surveyed

Age group (years)	Total participants	Gender	
	*n* (%)	Male (*n*)	Female (*n*)
5–16	157 (38)	67	90
17–24	72 (17)	32	40
25–44	119 (29)	48	71
≥ 45	67 (16)	36	31

### Iodine and selenium concentrations in edible plants and water

Plant samples were divided into six groups: leafy vegetables (*n* = 56), grains (*n* = 55), crop vegetables (*n* = 75), tubers (*n* = 30), fruits (*n* = 63) and nuts (*n* = 2). The concentrations of I and Se in all plant species (Tables 2 and 3, respectively) decreased in the order: leafy vegetables > grains > crop vegetables > tubers > nuts > fruits. Iodine and Se concentrations across all water samples ranged from 0.01 to 10 µg/L and 0 to 3.0 µg/L with median values of 0.24 (IQR 0.12, 0.72) and 0.27 (IQR 0.11, 0.46) µg/L, respectively (supplementary material Table B1).

**Table 2:** Iodine concentration (µg/kg) in edible plant groups and species (dry weight) collected in districts of Gilgit-Baltistan with comparison to other reported values

Plant group/species	No of samples	Current study		Literature data
		Mean ± SD	Range	Mean iodine concentration
**Crop vegetables**	75	16.9 ± 19.8	0.79–142	130^a^, 30^b^
Aubergine/egg plant	7	10.6 ± 8.71	4.75–29.3	306^c^
Beans	11	5.66 ± 3.47	1.42–11.4	10^d^, 70^d^, 186^e^
Capsicum	3	7.57 ± 2.83	4.35–9.68	157^e^, 394^c^, 351^f^
Chilli	11	15.4 ± 7.87	7.43–30.7	110^d^, 210^d^, 201^e^
Cucumber	5	14.5 ± 6.30	5.21–20.5	195^e^, 231^c^
Okra	10	33 ± 26.2	15.0–104	110^d^, 210^d^, 255^c^, 450^f^
Pigeon pea	1	0.79		10^d^
Pumpkin	6	15.6 ± 8.25	4.07–24.7	10^d^
Tomato	21	21.1 ± 28.2	5.01–142	20^d^, 30^d^, 80^d^, 65.1^e^, 513^c^, 150^f^
**Fruits**	63	6.51 ± 4.35	1.26–32.4	18^a^, 71.6^e^, 40^b^
Apple	18	5.38 ± 3.01	1.26–12.4	53^f^
Apricot	19	5.65 ± 1.83	3.35–11.8	
Ber/Chinese date	1	12.0		
Fig	3	8.14 ± 4.56	5.05–13.4	
Grapes	1	32.4		960^f^, 24.3^g^
Mulberry	3	11.4 ± 2.10	9.80–13.7	
Peach	4	5.45 ± 1.88	3.86–7.62	
Pear	11	6.64 ± 1.91	4.07–9.78	62^e^
Persimmon	3	2.73 ± 0.89	1.74–3.47	
**Grains**	55	21.8 ± 11.0	1.18–49.8	6.1^h^, 96.5^e^, 13^d^
Barley	3	33.9 ± 2.91	30.8–36.5	90^i^, 48.9^e^, 9^j^
Buckwheat	2	10.0	9.97–10.1	
Maize/corn	25	24.3 ± 7.97	9.16–49.8	91^i^, 10^d^, 80^ k^, 330^ k^, 122^e^, 20.3^l^
Wheat	25	18.7 ± 12.7	1.18–44.2	35^a^, 13^m^, 15^n^, 6.89^ h^, 227^i^, 74.4^e^, 12.5^c^, 40^j^, 17^l^
**Leafy veg**	56	36.3 ± 30.2	3.05–109	236^a^, 316^e^
Amaranth leaves	14	26.1 ± 16.4	6.17–63.8	163^f^, 70^d^, 160^d^
Cabbage	7	5.51 ± 1.97	3.05–8.75	20^d^, 80^d^, 123^e^
Kholrabi	13	17.2 ± 4.43	12.7–27.4	49^e^
Lettuce	1	107		455^e^
Spinach	21	61.8 ± 27.5	28.3–109	660^e^, 462^c^, 370^ l^
**Nuts**	2	6.62	2.52–10.7	218^a^, 126^e^, 250^b^
Almond	1	10.7		
Walnut	1	2.52		
**Tubers**	30	9.96 ± 11.5	2.94–66.4	8^d^
Carrot	4	12.6 ± 8.22	6.67–24.2	143^e^, 25^j^
Onion	12	11.1 ± 17.5	2.94–66.4	10^g^, 143^e^, 145^c^
Potato	11	7.77 ± 2.77	5.28–13.2	16^a^, 82^n^, 103^e^, < 10^j^, 190^f^, 70^b^
Radish	1	16.0		224^c^
Turnip	2	6.81	5.43–8.19	333^e^

^a^Haldimann et al. (2005); ^b^Johnson (2003); ^c^Karim (2018); ^d^Watts et al. (2015); ^e^Fordyce (2003); ^f^Mahesh et al. (1992); ^g^Salau et al. (2011); ^h^Shinonaga et al. (2001); ^i^Aquaron et al. (1993); ^j^Johnson et al. (2002); ^k^Longvah and Deosthale (1998); ^l^Eckhoff and Maage (1997); ^m^Zia et al. (2014); ^n^Anke et al. (1993)

**Table 3:** Selenium concentration (µg/kg) in edible plant groups and species (dry weight) collected in districts of Gilgit-Baltistan with comparison to other reported values

Plant group/species	No of samples	Current study		Literature data
		Mean ± SD	Range	Mean selenium concentration
**Crop vegetables**	75	34.2 ± 132	0.782–1140	118^a^
Aubergine/egg plant	7	8.62 ± 2.65	4.16–12.2	60.3^b^, 28^c^, 67^d^
Beans	11	116 ± 339	1.43–1137	210^a^, 56^e^, 31^f^
Capsicum	3	3.46 ± 1.49	2.23–5.11	59.2^b^, 52^d^
Chilli	11	10.9 ± 10.0	0.782–29.1	
Cucumber	5	19.8 ± 20.0	8.10–54.9	95.1^b^, 72.2^g^, 21^d^
Okra	10	25.9 ± 58.8	0.938–191	37.6^b^, 27^d^
Pigeon pea	1	19.4		60^e^, 150^h^
Pumpkin	6	20.9 ± 15.2	3.11–37.6	11.9^g^, 12^d^
Tomato	21	28.5 ± 23.1	4.28–77.9	108^b^, 36.2^g^, 39^c^, 15^d^
**Fruits**	63	4.40 ± 3.47	0.741–14.9	1.2^i^
Apple	18	4.10 ± 2.79	1.15–10.3	3^i^, 4.5^j^, 38^c^, 11^d^
Apricot	19	4.26 ± 2.73	1.09–13.1	
Ber/Chinese date	1	14.0		
Fig	3	8.13 ± 3.69	4.21–11.5	32^d^
Grapes	1	14.9		23^d^
Mulberry	3	5.64 ± 0.92	4.66–6.50	
Peach	4	1.49 ± 0.47	0.80–1.74	25^c^
Pear	11	3.63 ± 3.76	0.740–11.7	20^c^
Persimmon	3	2.02 ± 1.0	1.43–3.17	
**Grains**	55	23.8 ± 27.0	1.38–132	27.8^a^
Barley	3	26.4 ± 17.7	9.40–44.8	35.5^a^, 500^l^, 329^h^, 193^m^, 54.8^g^, 220^c^, 165^d^
Buckwheat	2	16.2	13.4–18.9	
Maize/corn	25	19.0 ± 17.8	1.38–55.2	4.6^a^, 149^h^, 49.4^b^, 110^d^
Wheat	25	28.9 ± 35.2	2.04–132	400^l^, 625^h^, 69^d^, 70.5–74.1^h^
**Leafy veg**	56	105 ± 195	1.17–1020	
Amaranth leaves	14	48.6 ± 51.5	13.9–218	362^h^
Cabbage	7	202 ± 368	1.17–1020	38^h^, 46^g^, 31^c^, 9^d^
Kholrabi	13	164 ± 248	2.64–807	37^g^
Lettuce	1	31.1		101^g^, 10^d^
Spinach	21	77.4 ± 127	4.16–536	127^h^, 117^b^, 169^g^, 7^d^
**Nuts**	2	28.5	4.01–52.9	295^a^
Almond	1	4.01		92^a^, 15^d^, 15^n^
Walnut	1	52.9		406^a^, 31^n^
**Tubers**	30	12.9 ± 14.0	1.49–45.7	
Carrot	4	12.5 ±	1.49–38.2	43.4^g^, 2^d^
Onion	12	12 ±	2.25–45.7	15°, 20^l^, 180^ h^, 55.3^b^, 40.9^g^, 137^c^, 43^d^
Potato	11	10 ±	1.89–37.5	30^e^, 30^l^, 28^i^, 325^h^, (30–70)^j^, 28.4^g^, 48^c^
Radish	1	24		95.9^b^, 18^g^, 13^c^, 33^d^
Turnip	2	29.6	29.2–30.1	29.1^g^, 20^c^

^a^Díaz-Alarcón et al. (1996); ^b^Karim (2018); ^c^Waheed et al. (2002); ^d^Al-Ahmary (2009); ^e^Arthur (1972); ^f^Simonoff et al. (1988); ^g^DeTemmerman et al. (2014); ^h^Singh and Garg (2006); ^i^Olson and Palmer (1984); ^j^Navarro-Alarcon and Cabrera Vique (2008); ^k^Mangan et al. (2016); ^l^Askar and Bielig (1983); ^m^Hussein and Bruggeman (1999); ^n^Barclay et al. (1995); ^o^Bratakos et al. (1987)

### Salt iodine concentration

Iodine concentration in salt samples ranged from 0.03 to 45.8 mg/kg (median 4.2; IQR 2.99, 10.8) and was not normally distributed (*p* < 0.005) even when log-transformed (supplementary material Fig. A1). There were five different brands of salt available locally, but all lacked information on I concentration or species (iodide or iodate).

### Questionnaire

The household (*n* = 76) questionnaire showed 85% (*n* = 65) of households use iodised salt; the majority (95%) stored salt in lidded containers. Based on the amount of salt *purchased*, per capita salt use was estimated as 52.8 g/day.

### Urinary sodium concentration (UNaC)

The median UNaC, uncorrected (UNaC_uncor_), corrected for creatinine (UNaC_cre_) or specific gravity (UNaC_sg_) were 133, 198 and 143 mmol/L, respectively (Table 4), which are equivalent to 186, 277 and 200 mmol/day based on a urinary volume of 1.4 L in 24 h (Karim, 2018; Medlineplus, 2019). From UNaC, it was estimated that the median salt intake in the study area was 12, 18 and 13 g/day based on UNaC_uncor_, UNaC_cre_ and UNaC_sg_, respectively.

**Table 4:** Summary statistics for urinary Na concentration (UNaC): uncorrected urinary Na (UNaC_uncor_); creatinine-corrected urinary Na (UNaC_cre_); specific gravity-corrected urinary Na (UNaC_sg_) for different age groups in the population that was surveyed in Gilgit-Baltistan, Pakistan, during winter 2018

Age group (years)	Sample population “*n* (%)”	UNaC_uncor_ (mmol/L)		UNaC_cre_ (mmol/g)		UNaC_sg_ (mmol/L)	
		Median (IQR)	Range	Median (IQR)	Range	Median (IQR)	Range
All age groups	415 (100)	133 (89, 180)	6.19–395	198 (115, 305)	4.38–3600	143 (104, 186)	5.63–341
5–16	157 (38)	134 (82, 176)	8.10–395	247 (148, 377)	6.49–3600	148 (107, 192)	19.9–296
17–24	72 (17)	140 (101, 194)	53.2–376	172 (111, 237)	31.7–612	145 (107, 182)	39.9–284
25 – 44	119 (29)	137 (89, 192)	8.29–367	178 (108, 280)	4.38–2020	147 (100, 195)	5.63–341
≥ 45	67 (16)	119 (81, 153)	6.19–246	141 (108, 291)	11.6–2850	118 (83, 168)	12.6–315
WRA (15–49)	134 (32)	130 (87, 179)	8.29–376	168 (110, 264)	4.38–981	140 (98, 177)	5.63–341

WRA = women of reproductive age

### Urinary iodine concentration (UIC)

Figure A2 (supplementary material) shows the distribution of uncorrected UIC (UIC_uncor_), creatinine-corrected UIC (UIC_cre_) and specific gravity-corrected UIC (UIC_sg_). The median UIC_uncor_ for all participants (*n* = 415) was 78 µg/L, similar to median UIC_sg_ (83 µg/L) but smaller than UIC_cre_ (114 µg/L) (Table 5). No significant difference was observed between the UIC of male and female participants at the household level (*p* > 0.05). No correlation (*p* > 0.05) was observed between UIC values and age for all participants together. The median concentration of UIC_cre_ differed across the age groups while those of UIC_uncor_ and UIC_sg_ were consistent (Table 5).

**Table 5:** Summary statistics for urinary iodine concentration (UIC) (uncorrected urinary I; UIC_uncor_, creatinine-corrected urinary I; UIC_cre_, specific gravity-corrected urinary I; UIC_sg_) in the population that was surveyed in Gilgit-Baltistan, Pakistan, during winter 2018

Age group (years)	Sample population "*n* (%)"	UIC_uncor_ (µg/L)		UIC_cre_ adjusted (µg/g)		UIC_sg_ adjusted (µg/L)	
		Median (IQR)	Range	Median (IQR)	Range	Median (IQR)	Range
All age groups	415 (100)	78 (43, 134)	5.87–773	114 (68, 188)	6.05–2120	83 (54, 127)	9.52–631
5–16	157 (38)	76 (41, 134)	5.87–696	128 (88, 218)	6.05–1560	84 (53, 129)	9.52–631
17–24	72 (17)	80 (52, 133)	14.5–566	99 (56, 149)	22.1–767	80 (55, 117)	17.0–386
25–44	119 (29)	83 (46, 139)	8.94–773	111 (70, 166)	28.0–1330	89 (61, 129)	27.4–567
≥ 45	67 (16)	66 (40, 115)	10.1–442	98 (60, 187)	27.8–2120	76 (45, 109)	12.8–538
WRA (15–49)	134 (32)	67 (46, 124)	8.94–773	96 (59, 157)	22.2–811	70 (54, 113)	17.0–567

WRA = women of reproductive age

### Urinary selenium concentration (USeC)

Figure A3 (supplementary material) shows the distribution of USeC of all participants together (*n* = 415). The median USeC_uncor_ was 17 µg/L, similar to the median USeC_sg_ (17 µg/L) but smaller than USeC_cre_ (23 µg/L) (Table 6). No significant difference was observed between the USeC of male and female participants at the household level (*p* > 0.05). There was no correlation (*p* > 0.05) between age (whole dataset) and USeC. As expected from UIC, the median concentration of USeC_cre_ differed across the age groups from that of USeC_uncor_ and USeC_sg_, both of which were consistent (Table 6).

**Table 6:** Summary statistics for urinary Se concentration (USeC) (uncorrected urinary Se; USeC_uncor_, creatinine-corrected urinary Se; USeC_cre_, specific gravity-corrected urinary Se; USeC_sg_) in the different age group of the population that was surveyed in Gilgit-Baltistan, Pakistan, during winter 2018

Age group (years)	Sample population "*n* (%)"	USeC_uncor_ (µg/L)		USeC_cre_ (µg/g)		USeC_sg_ (µg/L)	
		Median (IQR)	Range	Median (IQR)	Range	Median (IQR)	Range
All age groups	415 (100)	17 (10, 24)	0.948–73.1	23 (18, 31)	1.07–398	17 (13, 21)	1.5–53
5–16	157 (38)	16 (10, 23)	1.33–61.3	28 (23, 35)	1.07–220	18 (14, 21)	5.3–41
17–24	72 (17)	19 (12, 27)	3.93–62.7	20 (16, 24)	6.09–40.1	16 (13, 22)	8.2–30
25–44	119 (29)	17 (10, 27)	0.948–73.1	20 (15, 28)	2.97–212	16 (13, 20)	5.5–49
≥ 45	67 (16)	17 (9, 24)	1.19–56.1	22 (17, 29)	10.3–398	17 (12, 22)	1.5–53
WRA (15–49)	134 (32)	17 (11, 27)	0.948–55.1	21 (18, 28)	2.97–97.0	16 (13, 21)	5.5–41

*WRA* women of reproductive age

### Hydration correction

**Fig. 2:**
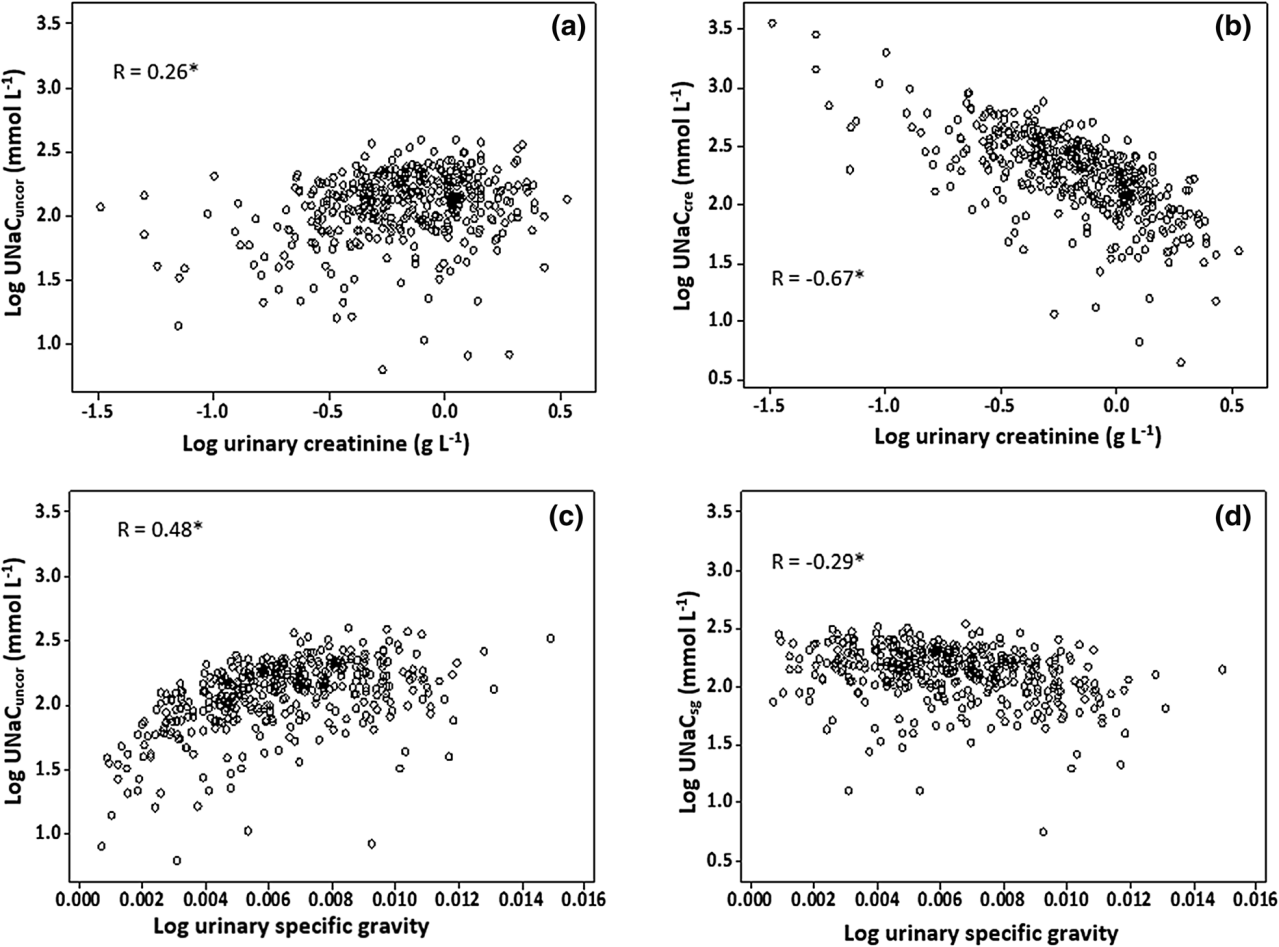
Scatter plot of urinary sodium concentration (uncorrected—UNaC_uncor_; creatinine-corrected—UNaC_cre_; specific gravity-corrected—UNaC_sg_) against dilution measurements. *Denotes statistical significance of Pearson correlation to *p* < 0.001

**Fig. 3:**
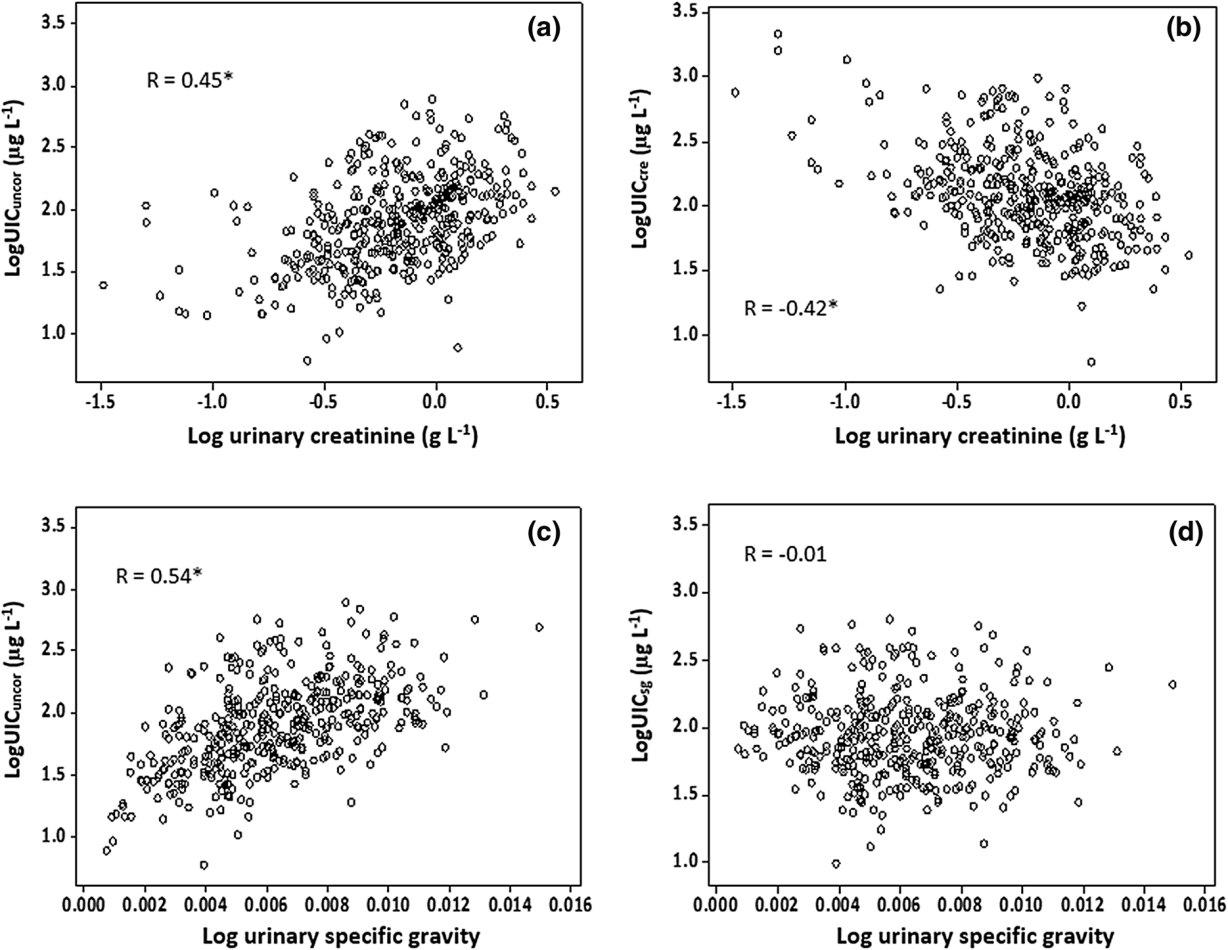
Scatter plot of urinary iodine concentration (uncorrected—UIC_uncor_; creatinine-corrected—UIC_cre_; specific gravity-corrected—UIC_sg_) against dilution measurements. *Denotes statistical significance of Pearson correlation to *p* < 0.001

**Fig. 4:**
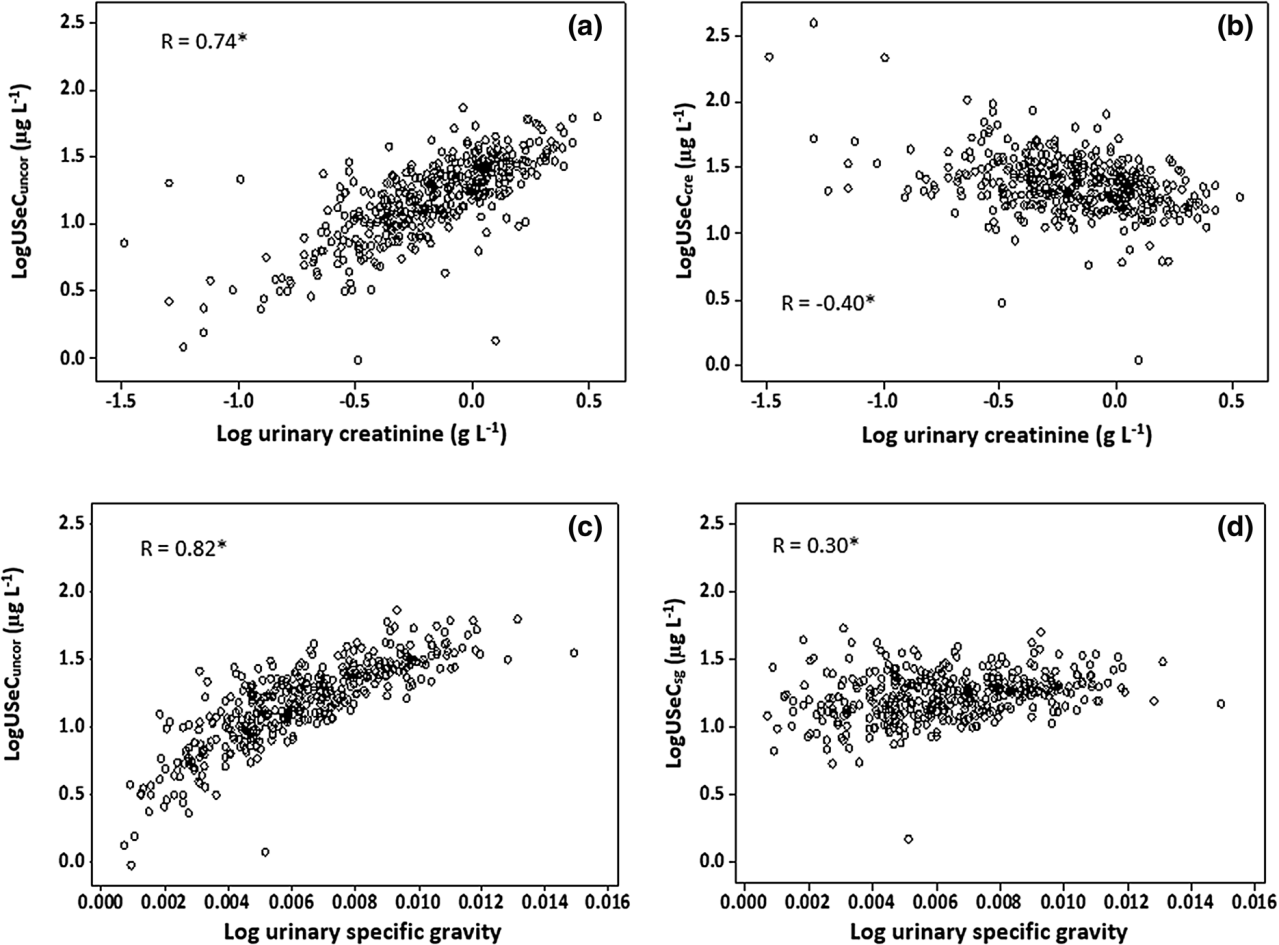
Scatter plot of urinary selenium concentration (uncorrected—USeC_uncor_; creatinine-corrected—USeC_cre_; specific gravity-corrected—USeC_sg_) against dilution measurements. *Denotes statistical significance of Pearson correlation to *p* < 0.001

Uncorrected concentrations of analytes (Na, I and Se) showed positive correlations with both creatinine and specific gravity (Figs. 2, 3 and 4) suggesting the need for correction to remove hydration-driven variation. The effectiveness of correction is seen in the reduced correlation in corrected data (Figs. 2, 3 and 4). A greater proportion of variation in UIC was attributable to sample dilution, as shown by specific gravity correction, and was less evident for creatinine correction (Figs. 2, 3 and 4).

### Iodine and selenium daily dietary intake

Daily I and Se intakes, based on a typical Pakistani national food basket, were estimated as 46.4 and 32.3 µg/day, respectively, using a combination of measured I and Se concentrations in locally grown foods and literature values for the remaining food items (Table 7). The I and Se intakes from drinking water were 1.28–2.56 and 0.78–1.17 µg/day, respectively, based on 2–4 L daily consumption. Combined estimated intakes of I and Se from food and water were therefore 47.7–49.0 and 33.1–33.5 µg/day, respectively. Comparison between reported I and Se contents of foods in the wider literature with the values measured for locally grown produce in this study indicates that locally sourced foods are typically lower in I and Se (Tables 2 and 3). The use of literature values may therefore overestimate I and Se intake. If, instead, we scale the intakes measured for the local foods (65% of the diet; I = 8.8 µg/day and Se = 17.7 µg/day) to 100%, then I and Se intake is estimated as 14.1–15.7 and 28.0–28.4 µg/day, respectively.

**Table 7:** An estimate of individual adult daily I and Se intake from dietary sources calculated based on estimated average requirements of 95 µg/day I and 45 µg/day Se

Food item	Consumption (g/day)	I concentration (µg/kg)	Se concentration (µg/kg)	Dietary I intake (µg/day)	Dietary Se intake (µg/day)	% EAR of I	% EAR of Se
Wheat*	350	18.7	28.9	6.55	10.1	6.89	22.4
Rice**	60	14.7	100	0.882	6.00	0.928	13.3
Other cereals*	15	24.3	19.5	0.365	0.293	0.384	0.651
Pulses*	30	5.26	108	0.158	3.24	0.166	7.20
Meat products**	40	183	98.9	7.32	3.96	7.71	8.80
Dairy products**	150	195	31.2	29.3	4.68	30.8	10.4
Fruits and vegetables*	100	17.8	40.6	1.78	4.06	1.87	9.02
				46.4	32.3	48.8	71.9

*I and Se concentration values obtained from samples analysed in the current study

**I and Se concentration values obtained from the literature (*Sources*: Hussain, 2001; Fordyce, 2003; Iqbal et al., 2008; Karim, 2018; USDA, 2019)

### Iodine and selenium nutritional status

Considering the WHO-recommended concentrations for UIC, 63% of the Gilgit-Baltistan sampled population had UIC_uncor_ < 100 µg/L and 6% had UIC_uncor_ > 300 µg/L_._ UIC_cre_ indicated only 42% participants with < 100 µg/L and 13% > 300 µg/L while for UIC_sg_ 64% were < 100 µg/L and 6% > 300 µg/L. We can make no comment on pregnant and nursing women as their numbers were too small to be considered separately.

For Se, compared to the ‘Estimated Average Requirement’ (EAR) (IOM, 2000), a higher proportion (46–90%) of individuals in age groups ≥ 14 years had insufficient Se intake based on USeC_uncor_ and USeC_Cre_ while USeC_cre_ indicated that 14–68% individuals had insufficient Se intake (Table 8). The mean Se intake in pregnant and lactating women was ≤ 54 µg/day estimated from USeC (USeC_uncor_; USeC_cre_; USeC_sg_) assuming that 50–70% of dietary Se is excreted in urine.

**Table 8:** Number and percentage of individuals with inadequate Se intake according to age groups

Age groups (year)	No of participants	Se EAR (µg/day)	Number (%) of individuals with inadequate Se intake estimated from uncorrected and corrected USeC		
			USeC_uncor_	USeC_cre_	USeC_sg_
4–8	50	23	9(18)–13 (26)	1 (2)–1 (2)	1 (2)–4 (8)
9–13	69	35	25 (36)–39 (57)	1 (1)–5 (7)	16 (23)–35 (51)
14–18	66	45	31 (47)–46 (70)	9 (14)–35 (53)	30 (46)–53 (80)
19–50	199	45	91 (46)–127 (64)	60 (30)–119 (60)	94 (47)–159 (80)
> 51	31	45	15 (48)–23 (74)	2 (7)–21 (68)	15 (48)–28 (90)

The age groups and estimated average requirement (EAR) of Se is based on Institute of Medicine recommended reference values. The number of individuals in different age groups provided in this table had daily Se intake less than their respective RDA estimated based on the loss of 50–70% dietary Se in urine

### Quality control recoveries

The recoveries of I and Se in the wheat flour and tomato leaf CRMs are provided in supplementary material Table B2. The recoveries (%) of I in the urine CRMs Seronorm L-1 (certified value: 105 µg/L, measured value: 98.7 ± 2.9 µg/L, *n* = 4) and Seronorm L-2 (certified value: 297 µg/L, measured value: 297 ± 9.7 µg/L, *n* = 4) were 94% and 100%, respectively.

## Discussion

This study is the first investigation to evaluate local population I and Se status in NE Pakistan in relation to their dietary intake and to use urine as a biomarker for population Se status. It is apparent that the Gilgit-Baltistan population is at risk of both dietary I and Se deficiency.

There is no recommended range of I concentrations in drinking water for human consumption (WHO, 2008), but the concentrations found in Gilgit-Baltistan (0.01–10 µg/L) are at the bottom of the internationally reported ranges. Drinking water I concentration ranges of 0.2–304, < 1–139 and 55–545 µg/L have been reported in Kenya, Denmark and Algeria respectively (Watts et al., 2019). Selenium concentration in drinking water (0–3.0 µg/L) was also at the lower end of the global range, of 0.06 to > 6000 µg/L (WHO, 2011). However, Se concentration in public water supplies usually does not exceed 10 µg/L (WHO, 2011).

Crop types in Gilgit-Baltistan typically had lower I and Se concentrations when compared to published values for crops from other areas (Tables 2 and 3), possibly due to the low concentrations of iodine and selenium in soil available for plant uptake (Ahmad et al., 2021). Gilgit-Baltistan is a remote mountainous area, where the population largely consumes locally grown agricultural produce. Assuming a typical Pakistani national food basket (Hussain, 2001), the consumption of locally produced food items would only provide 49% and 72% of I and Se EAR based on concentrations in this study and the literature (Hussain, 2001; Fordyce, 2003; Iqbal et al., 2008; USDA, 2019). If local food is used to calculate daily intake, then the diet would provide only 9% and 39% of I and Se EAR, respectively. The daily intake of I and Se from drinking water would be < 3% of I and Se EAR. This is much smaller than the I contribution (10%) from drinking water suggested by Gao et al., (2021). The smaller contribution of Se from drinking water is not unusual as most drinking water has < 10 µg/L of Se (WHO, 2011). Thus, estimated intakes of I and Se in Gilgit-Baltistan fall substantially below recommended levels.

Iodised salt is a proven approach to the prevention of IDD. The I concentration in the majority of Gilgit-Baltistan salt samples (83%) was well below the minimum level of the WHO-recommended range of 20–40 mg I per kg salt (WHO/UNICEF/ICCID, 2007). Our results are comparable with the fortification assessment coverage toolkit survey undertaken in 2017 in Pakistan in which 87% of different iodised salt brands (*n* = 30) collected from marketplaces across Pakistan were shown to be inadequately iodised (IGN, 2020c). Our observed salt I concentration was lower than that reported in the 2011 national nutrition survey: 30% of Gilgit-Baltistan iodised salt had ‘inadequate’ I, using 15 mg I per kg of salt as the ‘adequate’ threshold (GoP, 2011). The reasons for this are not known. However, possible causes may include initial inadequate addition of I, poor mixing and/or storage conditions which may affect volatilisation (light, humidity, temperature). Most households stored salt in lidded containers at home, which may help to reduce salt moisture content and volatilisation of I. In the current study, no information was collected from participating households on the storage time of the salt and there was no indication of dates of production on the manufacturers’ labels. Iodine loss of 8.4–23% from different types of iodised salts, stored in different lidded containers over 15 days, has been reported (Jayasheree & Naik, 2000); the stability of I in iodised salt stored at room temperature at a relative humidity of 30–45% was 59% after ~ 3.5 years (Biber et al., 2002). Further investigations are required to establish the reasons for such low salt I concentrations which put at risk the health of the local community and challenge the integrity of the national salt iodisation programme.

The widespread use (85%) of iodised salt in the region demonstrates that either the communication of the need for iodised salt has fully penetrated this remote region, or that the supply of non-iodised salt has been reduced significantly. Either reason produces a good result for the national iodised salt programme. The current use of iodised salt at household level (85%) was slightly lower than previously reported (91%) for Gilgit-Baltistan but higher than the national average (80%) and the average for rural areas of Pakistan (GoP/UNICEF, 2018). Based on the amount of salt *purchased* by each household, per capita salt use was estimated as 52.8 g/day, which is far greater than normal salt *consumption* of 5 to 15 g/day across different cultures (Medeiros-Neto & Rubio, 2016). This is also ten times greater than the WHO-recommended salt intake of 5 g/day (WHO, 2012). The household salt intake estimation from monthly purchases is therefore wholly unreliable. There may be loss of salt through spillage, in cooking water and from the use of salt for other purposes such as preserving meat, particularly during the religious festival of Eid ul Adha when animals are slaughtered.

About 85–90% of Na ingested is excreted in urine (WHO, 2007; Cogswell et al., 2018). Hence, daily salt intake estimated from UNaC is more reliable than that calculated from monthly or weekly salt purchase. However, UNaC still underestimates the actual salt intake by about 10–15% as it only accounts for electrolyte loss via kidney and excludes other forms of excretion (Amara & Khor, 2015). High salt (Na) intake is associated with adverse health effects including high blood pressure and cardiovascular diseases (Cappuccio, 2013; Partearroyo et al., 2019). The salt intake estimated by UNaC_cre_ (18.0 g/day) was higher than that predicted by UNaC_uncor_ (12.0 g/day) or UNaC_sg_ (13.0 g/day). The salt intake calculated from UNaC_uncor_ or UNaC_sg_ fell within the normal range for daily salt consumption (5–15 g) but was much lower than the consumption rate estimated from the salt intake questionnaire survey. However, it still indicates a higher salt consumption than the WHO-recommended amount of 5 g/day (WHO, 2012). It is also slightly higher than the average global salt intake of 10.1 g/day (Powles et al., 2013). However, the salt intake estimated from UNaC (UNaC_uncor_, UNaC_sg_) is comparable with salt consumption reported in other countries in the region such as Bangladesh and India, which have a similar cuisine. A daily salt consumption of 13.4 g (UNaC) was reported for Bangladesh (Zaman et al., 2017) and 11 g (24-h recall) in India (Johnson et al., 2019). The daily salt consumption found for Gilgit-Baltistan is higher than other countries; for example, UNaC-based salt consumption rates of 6.5, 8.0, 8.3, 8.4 and 10 g/day have been reported for Malaysia, the UK, Singapore, Thailand and Vietnam, respectively (Amarra & Khor, 2015; Bates et al., 2016). One of the reasons for higher salt intake could be the consumption of salty tea, a local tradition. Whether there is evidence of hypertension or other disorders associated with salt intake was not investigated.

Average daily iodised salt intake estimated from UNaC_uncor_ or UNaC_sg_ in Gilgit-Baltistan would provide ≤ 54 µg/day of I to an adult based on the median (4.2 mg/kg) I concentrations in locally available iodised salts. The combined I intake from all food sources and drinking water, together with the contribution from iodised salt, was approximately ≤ 103 µg/day; this is still below the RDA (150 µg/day). Therefore, we note that, although an iodised salt programme is running in the area, the local population may still be living at risk of I deficiency and the resulting disorders. If I contribution from iodised salt is considered based on the salt intake estimated from UNaC_cre_, then the total dietary I intake from all sources would be 124 µg/day (but see discussion of creatinine correction below) which is still below the RDA. This, of course, still leaves the population at risk from low Se intake, so the risk of IDD remains.

These estimates are put in context by the urinary I aspect of the study. According to the WHO, a population median UIC concentration of < 100 µg/L represents I deficiency and ≥ 300 µg/L shows an excessive I intake, which can also result in health complications, including hyperthyroidism and autoimmune thyroid diseases (Zimmermann & Andersson, 2012; WHO, 2013; Sun et al., 2014). The UIC concentrations (UIC_uncor_, UIC_sg_) confirm that the surveyed population have an inadequate I intake [mild deficiency (WHO, 2013)], leaving them at risk of a range of IDD. This is not surprising, given that I concentration was at the lower end of the global concentrations in the majority of locally grown agricultural produce and drinking waters, and that the population consumes locally grown food. Whether this deficiency is seasonal in Gilgit-Baltistan is unclear, given possible variations in a diet that largely depends on locally grown produce. The median UIC_cre_ values indicate sufficiency in some parts of the community (Table 5). However, there are reasons to doubt the creatinine correction.

The hydration correction with creatinine significantly influenced the rate of I deficiency or sufficiency in different age groups (Table 5), which is concerning since UIC_uncor_ and UIC_sg_ remained consistent across age groups. It is important to apply hydration correction to urinary analytes in order to compare data with other investigations, which may have examined populations with differing hydration statuses (Watts et al., 2015, 2019), which can distort comparisons between populations (Remer et al., 2006)—although WHO uses uncorrected UIC. The variation in UIC attributable to hydration status (dilution) is consistent with other investigations that creatinine correction is less useful as a corrective measure for hydration than specific gravity (Watts et al., 2019), despite the fact that specific gravity measurements made with a refractometer are prone to interference by urinary solutes such as protein, glucose and ketones (Middleton et al., 2016). Furthermore, creatinine excretion varies among demographic groups depending on protein intake, incidence of malnutrition, muscle mass, exercise, age and gender (Cockell 2015; Middleton et al., 2016; Watts et al., 2015, 2019). This would explain the differences found in our results (Table 5) between UIC_cre_ and UIC_uncor_ or UIC_sg_. In the context of this study, corrections with creatinine may be questionable due to the prevalence of malnutrition, with low protein intake within the Pakistani population (Aziz & Hosain, 2014; GoP/UNICEF, 2018). By contrast, correction with specific gravity may be a more reliable approach as it is also considered a proxy for osmolality—the most reliable approach for urinary dilution correction (Middleton et al., 2018, 2019; Watts et al., 2019). However, there are limited data available on, or discussion about, utilising alternative correction methods to creatinine. Standard guidelines on the proper use of dilution corrections are also lacking, and would benefit from further research. Nevertheless, we do not recommend the use of creatinine as a corrective factor in UIC surveys.

The daily Se intake estimated from USeC (USeC_uncor_, USeC_sg_) was below the EAR of 45 µg/day (IOM, 2000) in a large proportion (46–90% depending on method of evaluation) of the population. However, since UIC is a population measure we were unable to investigate how the I and Se intakes were related in any one individual. The fact that the creatinine-corrected urinary I concentration, if accepted as reliable, was above the WHO guideline value (Table 5) should not give rise to complacency, as the low Se status is likely to counteract this, adding to the picture of a community at risk of IDD.

Urinary Se excretion is one of the most effective indicators of Se intake (Rodríguez et al., 1995; Hawkes et al., 2003; Yoneyama et al., 2008) which suggest that USeC can be used to predict the Se intake of different populations (Longnecker et al., 1996; Nakamura et al., 2019). The median USeC for school-age children and women of reproductive age were comparable to or less than USeC values reported by Phiri et al. (2020) for a Malawian population, who were reported to be Se deficient based on their plasma Se status. A strong correlation (*r* = 0.962, *p* < 0.001) has been found between USeC and dietary Se intake over a wide range of diets from across the world (Alaejos & Romero, 1993). It is reported that approximately 50–70% of Se ingested is excreted in urine (Alaejos & Romero, 1993). Based on this approach, 24–81% of Gilgit-Baltistan participants aged ≥ 14 years were found to have inadequate Se intake, estimated from all measures of USeC. With the same approach, a comparatively smaller proportion of participants from age groups 4–8 and 9–13 years were found to be Se deficient by all measures of USeC. However, after hydration correction with creatinine the number of participants with inadequate Se intake decreased and this creatinine-driven effect was more pronounced in participants aged 4–8 and 9–13 years compared to participants in the ≥ 14 years age group. The reason for this could be the variation in creatinine concentration with age, sex and body weight, decreasing the reliability of the creatinine correction, again indicating the problems of the creatinine approach to hydration. We cannot recommend creatinine correction for hydration.

The USeC reflects Se intake and approximately 40–60% of Se intake is excreted in urine (EFSA NDA Panel, 2014), which, for the current study, means that approximately 32–54% participants of the surveyed population is living at risk of Se deficiency (USeC_uncor_, USeC_sg_) but 7–30% when USeC_cre_ is used. A study investigating daily Se intake in a Japanese population from 24-h urinary Se reported 73 and 77% of ingested Se excreted via urine in men and women, respectively (Nakamura et al., 2019; Yoneyama et al., 2008). If urine samples collected in our study are assumed equivalent to 24-h urine sampling, then the mean Se intake based on USeC_uncor_ and USeC_sg_ in males and females of all age groups ≥ 14 years would be less than the EAR (45 µg/day). Using USeC_uncor_ and USeC_sg_, participants aged 4–8 years have sufficient Se and those aged 9–13 years had an intake slightly below their EAR (35 µg/day). The intake estimated from USeC_cre_ indicated that the EAR of all age groups were met, but, as noted earlier, hydration correction with creatinine may not be very robust.

We conclude that Se intake in the majority of the Gilgit population appears to be low. However, further research is required to quantify Se status and daily Se intake from the consumption of different food items and its estimation from blood plasma and urinary Se in Gilgit. Currently, there are no accepted limits available to assess the severity of Se deficiency based on USeC; if these can be established, USeC could be used as an effective, non-invasive and cost-effective alternative to blood plasma assays.

No correlation (*p* > 0.05) was found between uncorrected and corrected UIC and USeC which conflicts with the findings of Karim (2018) who found a correlation between spot urine UIC_uncor_ and USeC_uncor_ (*n* = 410) in the Kurdistan region of Iraq. This also conflicts with the correlation between UIC_uncor_ and USeC_uncor_ in both spot and 24-h urine (*n* = 62) in a New Zealand population (Thomson et al., 1996). Our lack of correlation between UIC and USeC may indicate that these essential nutrients are supplied from different food sources in northern Pakistan.

This study is a snapshot in time, and this is not the ideal approach to describe I deficiency in a population because individual UIC can vary day to day or season to season as diet changes. The 2011 national nutritional survey of Pakistan noted that 76% of women (*n* = 34) from Gilgit-Baltistan and 48% nationwide (*n* = 1460) were deficient (GoP, 2011). In 2018, the national nutrition survey reported that 18% of women of reproductive age in Pakistan were I deficient (GoP/UNICEF, 2018). Our study suggests that I deficiency in this area is more common than reported in 2018. We included more participants from a more geographically confined area compared to the national nutrition survey and hence our work is more locally representative. Our findings on the I status of Gilgit-Baltistan women of reproductive age (median UIC of < 100 µg/L) are concerning, given the adverse outcomes of such deficiency on foetal brain development in particular (Toloza et al., 2020). Furthermore, the optimal I nutrition status in pregnancy and lactation is a median UIC within the range 150–249 µg/L_,_ indicating that children born to women entering pregnancy in Gilgit-Baltistan are likely to suffer reduced cognition (Velasco et al., 2018).

There was no correlation observed between salt intake and I status at a household or individual level (*p* > 0.05). The reason for this is not known, and it implies that iodised salt may not be the main source of I in the population of the study area, which is a disappointing result given the national iodised salt program. If iodised salt is the primary source of I for individuals, a relationship between UNaC and UIC should be apparent. Up to 90% of Na and I are excreted in urine and a good relationship has been noted between UIC and UNaC in a Kurdish population where iodised salt was the main source of I in the population (Karim, 2018).

Although the water and crop data indicate a limited supply of both I and Se from the local environment in Gilgit-Baltistan, there are no recognised standards to define an environment that is I deficient, whether for water, plants or soil. Environments may be classed as deficient simply by the local presence of IDD, or where the concentrations in water, crops or soils are small compared to other locations_._ An I concentration in water below 5–10 µg/L may indicate deficiency, but very few situations have been investigated using the full ‘source-pathway-receptor’ approach to demonstrate the deficiency pathway. While it is plausible, perhaps likely, that I concentrations in water, for example, below 5–10 µg/L contribute to the local IDD, this needs further proof since it is also plausible that other factors (goitrogens) antagonise the local I supply. On the one hand, there is clear evidence that increasing I intake decreases the incidence and prevalence of many IDD, but there are also many reports of the presence of local goitrogens (anti-thyroid agents) affecting the incidence and prevalence of IDD (Chandra et al., 2016; Eastman & Zimmermann, 2018; Gaitan, 1989; Langer & Greer, 1977; Mondal et al., 2016). In Gilgit-Baltistan, the I and Se intakes are both low. Selenium is necessary for the proper functioning of the deiodinase enzyme group and a low intake affects thyroid hormone function; Se itself is not goitrogenic, but low Se intake is. Thus, the aetiology of IDD in locations such as Gilgit-Baltistan, and other areas with low Se concentrations or the presence of known goitrogens, is likely to be more complex than can be confirmed simply from a low environmental I concentration, or even from a low (or normal) median UIC. This is an area ripe for research, since nowadays most communities with IDD are only mildly or moderately deficient in I, and any antagonism of thyroid hormone genesis and action may have a greater adverse effect on health than the UIC would predict. However, if not investigated such causes will be missed, with resulting difficulties in devising effective preventative programmes.

Strengths of the current study include analysis of I and Se content of dietary sources alongside related outcome measures at the population (I) and individual (Se) levels. The study had a large (*n* = 450) sample size of people from across the community, compared to previous studies with < 40 participants in Gilgit-Baltistan (GoP, 2011). We also compared, in this large dataset, different approaches to correction for hydration status and raise further questions about the utility of creatinine correction. Weaknesses include the small number of samples in some of the food classes (Table 2) and the lack of seasonal repetition of the work. Nor were we able to compare plasma Se with urinary Se concentrations to show robustly that our USeC results are reliable. Nevertheless, we do not believe that these weaknesses undermine the findings of the study. Despite all the difficulties and caveats, the fact remains that the Gilgit-Baltistan population remains at risk of I deficiency and the resulting range of IDD. There is an urgent need to improve the I nutrition of the population further than what has already been achieved.

## Conclusions

In summary, this study suggests that the Gilgit-Baltistan population is I deficient and at continuing risk of IDD’s. This status is compounded by Se deficiency, which can act as an antithyroid goitrogen, further decreasing the ability of the body to utilise the low I supply from the local diet. There is some uncertainty regarding hydration correction of UIC using creatinine in developing countries; correction with specific gravity may be more robust. Further research is required to establish proper guidelines for hydration correction of urinary analytes.

The problem of I deficiency could be resolved if the local/regional government can ensure adequate iodisation of salt and establish an annual UIC monitoring programme so that population iodine intake can be regularly checked. Furthermore, an awareness programme with the support of health professionals to encourage the intake of highly variable diet in women of childbearing age would also be useful in reducing the incidence of iodine deficiency. With regard to resolving Se deficiency, wheat biofortification with Se (selenate)-treated fertilisers would probably be the best solution as wheat is the primary staple carbohydrate crop in Gilgit-Baltistan and constitutes an essential and consistent item in all daily meals.

More work is needed to define the role of antithyroid substances (goitrogens) in I-deficient and borderline sufficient areas. Similarly, more work is needed to understand what environmental I concentrations are needed to supply adequate dietary I to the local community. It is not currently possible to infer population deficiency from any indices of ‘environmental iodine’. The use of the source-pathway-receptor approach would help achieve this objective.

## Supplementary Information

Below is the link to the electronic supplementary material.

Supplementary file1 (DOCX 5142 KB)

## Data Availability

The authors confirm that the summary of data supporting the findings of this study are available within the article. Detailed data is available from the corresponding author upon request.
