# Supplementary material for: Environmental and human iodine and selenium status: lessons from Gilgit-Baltistan, North-East Pakistan
Source: Environ Geochem Health. 2021 May 7;43(11):4665–86. doi: 10.1007/s10653-021-00943-w (PMC8528744; doi:10.1007/s10653-021-00943-w)
Supplement: Supplementary file 1 — Supplementary file1 (DOCX 5142 KB) [file 10653_2021_943_MOESM1_ESM.docx]

# Supplemental material


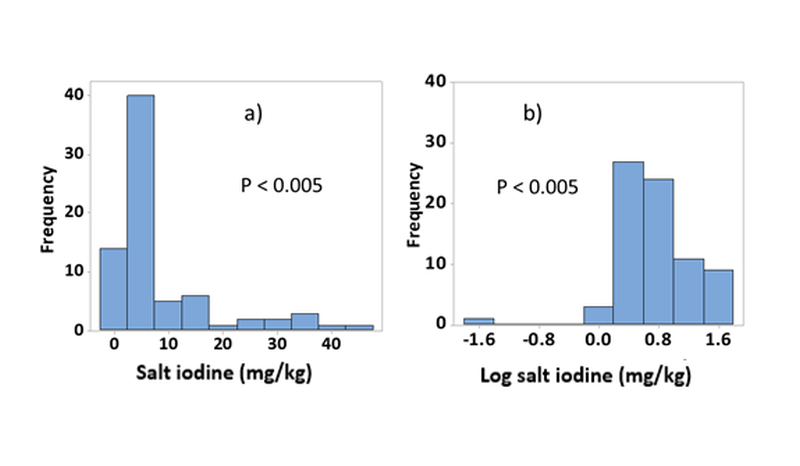


Fig. A1 Histograms of salt iodine concentration: a) numerical data; b) log-transformed data. The Anderson Darling normality test P value ≤ 0.05 indicates that data is not normally distributed.


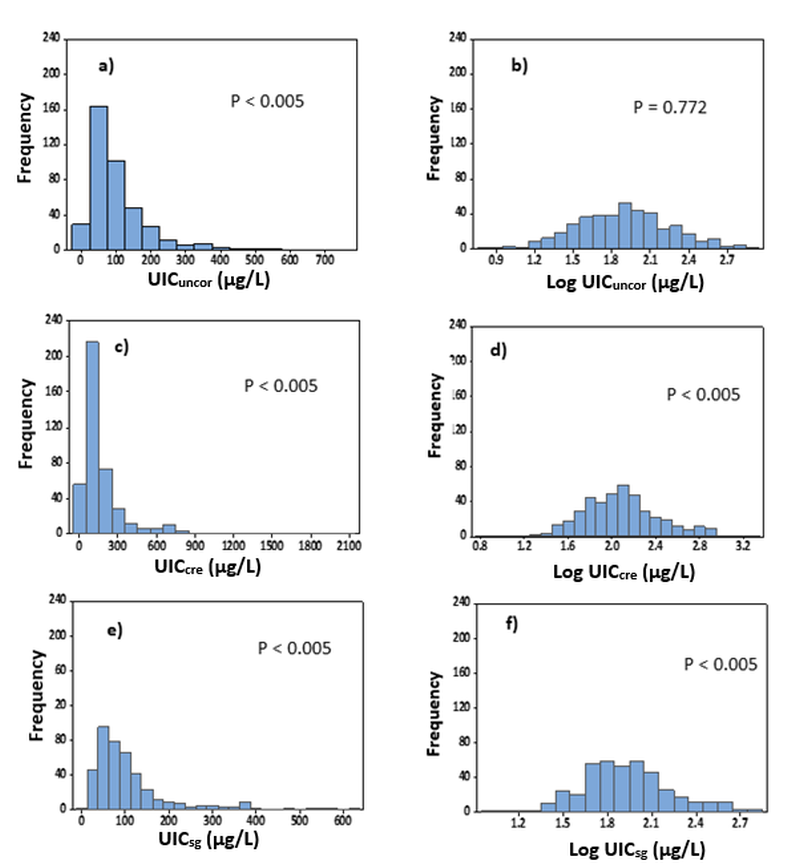


Fig. A2 Histograms of UIC: UIC_uncor_; UIC_cre_; UIC_sg_ numerical and log-transformed data. a) UIC_uncor_ numerical data; b) UIC_uncor_ log-transformed data; c) UIC_cre_ numerical data; d) UIC_cre_ log-transformed data; e) UIC_sg_ numerical data; f) UICsg log-transformed data. The Anderson Darling normality test P value ≤ 0.05 indicates that data is not normally distributed.


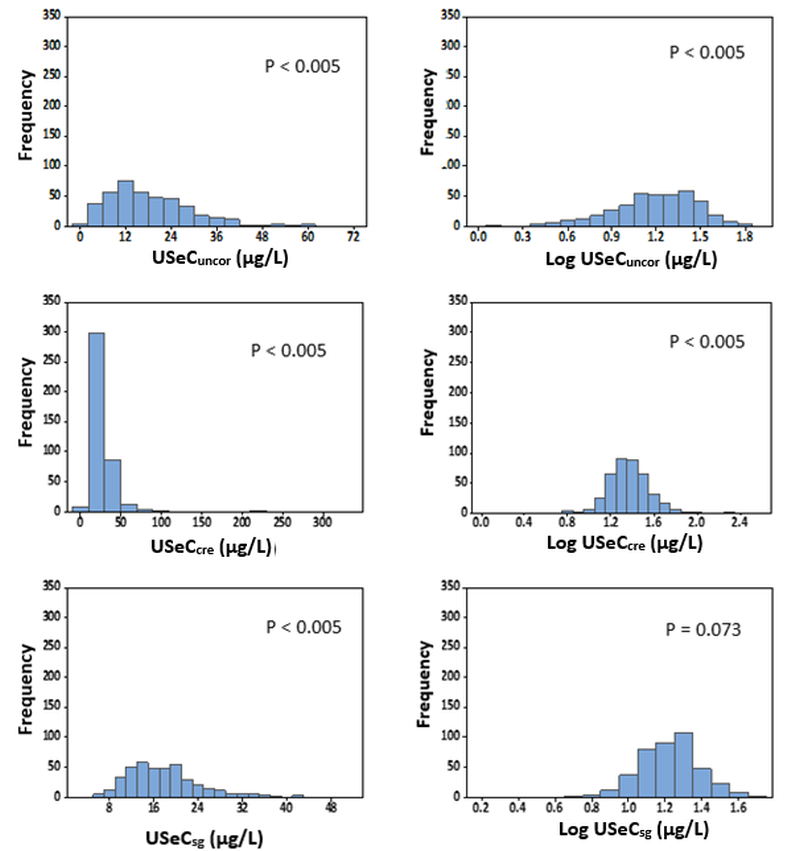


Fig. A3 Histograms of USeC: USeC_uncor_; USeC_cre_; USeC_sg_ numerical and log transformed data. a) USeC_uncor_ numerical data; b) USeC_uncor_ log transformed data; c) USeC_cre_ numerical data; d) USeC_cre_ log transformed data; e) USeC_sg_ numerical data; f) USeC_sg_ log transformed data. The Anderson Darling normality test P value ≤ 0.05 indicates that data is not normally distributed.

Table B1 Basic characteristics (mean ± SD) of water samples

| **Source type** | **Number**  **(n)** | **pH** | **EC** | **CaCO_3_ (equivalent)** | **Iodine** | **Selenium** |
| --- | --- | --- | --- | --- | --- | --- |
|  |  |  | **(µS/cm)** | **(mg/L)** | **(µg/L)** | |
| Surface water | 47 | 7.9 ± 0.37 | 172 ± 110 | 82 ± 51.6 | 0.28 ± 0.31 | 0.29 ± 0.37 |
| Groundwater | 35 | 8.0 ± 0.34 | 289 ± 206 | 155 ± 88.0 | 1.1 ± 1.8 | 0.54 ± 0.55 |

Table B2 Analytical results and recoveries of plant certified reference materials

| **Certified reference material (CRM)** | **Number (n)** |  |  | **Selenium** | **Iodine** |
| --- | --- | --- | --- | --- | --- |
| Wheat Flour  (NIST 1567b) | 6 | Measured | (mg/kg) | 1.17 ± 0.02 |  |
|  |  | Certified |  | 1.14 ± 0.10 |  |
|  |  | Recovery | (%) | 103 |  |
| Tomato leaves  (NIST 1573a) | 16 | Measured | (mg/kg) | 0.065 ± 0.002 | 0.73 ± 0.03 |
|  |  | Certified |  | 0.054 ± 0.003 | 0.85*^a^* |
|  |  | Recovery | (%) | 120 | 86 |

*^a^*Non certified value
